# Investigation into Changes of Microstructure and Abrasive Wear Resistance Occurring in High Manganese Steel X120Mn12 during Isothermal Annealing and Re-Austenitisation Process

**DOI:** 10.3390/ma15072622

**Published:** 2022-04-02

**Authors:** Mateusz Dziubek, Małgorzata Rutkowska-Gorczyca, Włodzimierz Dudziński, Dominika Grygier

**Affiliations:** 1 Faculty of Mechanical Engineering, Wroclaw University of Science and Technology, Wybrzeże Wyspiańskiego 27, 50-370 Wroclaw, Poland; malgorzata.rutkowska-gorczyca@pwr.edu.pl (M.R.-G.); dominika.grygier@pwr.edu.pl (D.G.); 2 Faculty of Technical and Economic Sciences, Witelon Collegium State University, Sejmowa 5A, 59-220 Legnica, Poland; wlodzimierz.dudzinski@pwr.edu.pl

**Keywords:** hadfield manganese steel, abrasive wear, isothermal heat treatment, grain refinement, M_3_C carbide

## Abstract

Hadfield steel, under unit pressure conditions, strengthens itself by forming a high density dislocation structure, which results in increased resistance to dynamic impact wear. However, under abrasion conditions, the homogeneous microstructure of the cast steel is insufficient to achieve the expected service life. The aim of the research is to conduct a comparative analysis of the material in its as-delivered state and after two-stage heat treatment (isothermal annealing followed by re-austenitisation). It was found that after isothermal annealing of X120Mn12 grade steel at a temperature of 510 °C, a microstructure with a complex morphology consisting of colonies of fine-grained pearlite, (Fe,Mn)_3_C carbides distributed along the grain boundaries of the former austenite and needle-like (Fe,Mn)_3_C carbides was obtained in the austenite matrix. The subsequent thermal treatment of the steel with the use of supersaturating annealing at 900 °C resulted in a heterogeneous microstructure consisting of evenly distributed globular carbide precipitations in a matrix of considerably finer austenite grains in comparison with the as-delivered original state. As a result of the final microstructural changes achieved, a 16.4% increase in abrasion resistance was obtained compared to the delivered condition.

## 1. Introduction

The conventional heat treatment for Hadfield steel is considered as subjecting the material to austenitisation, followed by the rapid cooling of the cast steel in water, and the saturating of the grains of the austenite γ solid solution. The austenitisation process is preferably preceded by slow heating of the material to a temperature range of 1010 to 1090 °C, and subsequently the annealing of it for a specified period of time (approximately 1 to 2 h for each 25 mm of the component’s cross-section [1,2]). Obtaining a homogeneous microstructure is considered to be the primary objective of thermal treatments carried out on the Hadfield cast steel used in commercial applications. Many applications of the material with this microstructure morphology confirm its reliability and safety, e.g., in open-pit mining as the working elements of jaw crushers. This procedure was developed and experimentally confirmed in a number of patents registered by R.A. Hadfield himself in the second half of the 19th and early 20th centuries [3]. Nowadays, this cast steel is widely used in components exposed to wear under high and dynamic unit pressure, which is due to its tendency of strain hardening generated during the crushing operation. Furthermore, the strengthening mechanism itself is of scientific interest to many research teams [4,5,6,7,8,9,10].

According to the developed heat treatment procedure, the expected resulting morphology is supersaturated homogeneous austenite with a grain size that is primarily determined by the casting process, i.e., the temperature and cooling speed of the casting [1]. However, the homogeneity of the microstructure does not favour abrasive wear resistance, and a material of analogous hardness with a non-homogeneous structure may have a significant difference in terms of the degree of wear [11]. Therefore, the development of a material that contains hard M_3_C type carbide precipitations in the metallic matrix is the main reason for the modification of the chemical composition of Hadfield cast steel by different research groups [12,13,14,15,16,17,18,19]. As modifiers for carbide formation and/or grain refinement, elements such as titanium [12,13,14,15,16], nitrogen [17], tungsten [14], vanadium [18], niobium [20] or aluminium [19] were used.

Simultaneously, attempts are being made to increase resistance to abrasive wear by increasing the carbon and manganese content. This translates into an increase in resistance to abrasive wear, but at the expense of a decrease in toughness. High-manganese steels with increased carbon content can only be used for low-impact abrasive conditions [21].

Research into the development of a two-step heat treatment represents a new approach to influence the microstructure and mechanical properties of high-manganese steels. It is composed of an isothermal annealing stage to produce numerous pearlite grains, which in a second step will undergo reconstruction during re-austenitisation, causing the steel to be recrystallised. This improves the mechanical properties compared to steels produced by single-step austenitising annealing [22,23,24].

An alternative heat treatment procedure for Hadfield steels can be achieved by long-term isothermal annealing in the temperature range of 260 °C to approximately 550 °C. This results in morphological changes in its microstructure, which is caused by the precipitation of carbides along grain boundaries and, depending on the conditions of the thermal process carried out, the decomposition of austenite into products of diffusion and/or non-diffusion transformations [1], which is directly related to the variation of Ms temperature values as a function of the carbon and manganese content [25]. The carbide precipitation process results in a depletion of carbon in the matrix, which consequently leads to an increase in the Ms temperature and a potential partial quenching of the cast steel. The microstructure, consisting of brittle carbides segregated along grain boundaries of austenite, as well as martensite needles within them, disqualifies the cast steel from being used in this condition under impact loading conditions.

However, reannealing cast steel offers the possibility of incomplete dissolution of carbides in the matrix, which, with the appropriate choice of heat treatment parameters, can fulfil a precipitation strengthening factor if its size and distribution do not critically affect impact toughness reductions. Moreover, the abrasion wear resistance will be increased, expanding the application possibilities of high-manganese steels, e.g., as more durable working elements for jaw crushers.

The heat treatment carried out and described in this article follows the trend of modifying high-manganese steel without the addition of carbide-forming elements. However, the selected heat treatment parameters and cooling conditions are designed to: (1) Avoid excessive austenite grain growth due to high temperature of solution heat treatment, which negatively affects mechanical properties [26,27]; (2) avoid the formation of micro-cracks [26,28] or the development of corrosion after the heat treatment [29] due to the use of salt solution in the cooling process; (3) maximise the proportion of pearlite colonies formation by long-term isothermal heating at relatively higher temperatures to sustain atomic diffusion.

The aim of the research was to conduct a comparative analysis of the material in its as-delivered and heat-treated condition. A two-stage heat treatment was carried out: (1) Isothermal annealing at 510 °C for twelve hours, and subsequent cooling with the furnace until an ambient temperature was reached; (2) re-austenitisation of steel at 900 °C.

## 2. Materials and Methods

As the test material, a sheet of high-manganese steel of a grade close to X120Mn12 with a thickness of 4 mm was used, which corresponds to Hadfield steel in terms of chemical composition. In order to determine the homogeneity of the material in the as-delivered condition, a series of tests was performed in accordance with the adopted test methodology, with the results being presented at the end of this section. Analysis of the material was carried out in the as-delivered condition.

Heat treatment of the material was performed using a laboratory muffle furnace FCF22SH from Czylok (Jastrzebie-Zdroj, Poland) by isothermally heating the Hadfield steel at a controlled temperature of 510 °C for twelve hours and then cooling it with a furnace to an ambient temperature. In addition, a re-annealing procedure was experimentally performed at 900 °C for fifteen minutes in order to observe any potential microstructural changes in the material.

The chemical composition of the as-delivered material was determined by optical emission spectroscopy using a Leco GDS-500A glow discharge analyser (St. Joseph, MI, USA). A series of replicates on the ground surface of the analysed material was performed at the outermost locations of the sheet in order to estimate the homogeneity of the chemical composition of the material for the experiment.

The morphology of the microstructure in the nital-etched state (5% solution) was observed using light and electron microscopy methods. It was analysed using a Phenom XL scanning electron microscope (Eindhoven, The Netherlands) and a Nikon Eclipse MA 200 light metallographic microscope (Tokyo, Japan) equipped with a Nikon DS-Fi5 CCD camera. SEM observations were conducted using material contrast (BSE detector, backscattered electrons) under 15 kV accelerating voltage conditions. The grain size was measured according to ASTM E112−13 [30].

Microanalysis of the chemical composition of the individual components of the structure was carried out using the EDX (energy dispersive X-ray analysis) method with a Phenom ProX scanning electron microscope (Eindhoven, The Netherlands) coupled to an X-ray microanalyser. The distribution of individual elements was performed by surface and linear microanalysis, in which the elements C, Mn, Si and Fe were identified.

Microstructure observations and the identification of individual phases in the heat-treated state were performed with a Hitachi H-800 transmission electron microscope (Tokyo, Japan) using the selected area electron diffraction (SAED) method. A 1-mm thick material sample was cut with a metallographic precision cutter and then mechanically thinned to a thickness of approximately 80 μm. A disk of φ3 mm in diameter was cut using a laboratory electro-spark erosion machine EDM MS534B (San Clemente, CA, USA), which was followed by electrochemical polishing using Struers TenuPol (Cleveland, OH, USA). The samples were polished at 100 mA/cm^2^ using a 9:9:2 solution of glacial acetic acid, 2-butoxyethanol and perchloric acid with a temperature of 10 °C.

The interpretation of the electron diffractograms was carried out using the DYFR software (created by Professor Włodzimierz Dudziński), which enabled theoretical images of electron diffractograms for the parameters defined according to the Pearson catalogue (a, b, c, α, β, γ) to be obtained [31,32,33]. The results achieved were then graphically compared (overlapping) with the registered images.

Hardness measurements were performed using the Vickers method in accordance with PN-EN ISO 6507-1:2018-5, taking a series of ten measurement points for each measurand to determine the standard deviation. Measurements with a load of 294.2 N (HV30) were made using a Zwick/Roell ZHU 187.5 hardness tester (Ulm, Germany), whereas the hardness measurements of the individual components making up the microstructure of the material after heat treatment were performed using a MMT-X7 microhardness tester (Akita, Japan), with loads of 9.807 N (HV1), 4.903 N (HV0.05), and 0.2452 N (HV0.025) being applied.

Simultaneously, the steel samples after isothermal annealing at 510 °C and re-austenitisation at 900 °C were subjected to comparative wear resistance tests in relation to their delivery condition with a T-07 tribotester (Radom, Poland) according to the requirements of GOST 23.208-79, as well as to observations of the wear surface morphology using a scanning electron microscope. According to the standard, the specimens are subjected to a weight measurement before and after the test, during which the loose abradant is applied between the surface of the specimen and a rubber wheel rotating at a constant speed and applied with a constant force of 44 N. The abrasive wear resistance coefficient was calculated according to GOST 23.208-79. Due to the identical nature of the test material, the coefficient is the ratio of the weight loss of the test material to the weight loss of the reference material.

## 3. Results and Discussion

### 3.1. Analysis of the Material in the As-Delivered Condition

The as-delivered material was subjected to chemical composition analysis by performing a series of ten measurement replicates at the outermost locations of the sheet (Table 1). The steel was found to be chemically equivalent to the L120G13 grade that is still in use in the Polish foundry industry—according to the withdrawn PN-H 83160:1988 standard [34], or similar to its European equivalent, X120Mn12 (1.3401), which is an unstandardised grade. The homogeneity of the material was also confirmed by a series of hardness measurements, with an average value of 200.0 ± 6.7 HV30 being obtained.

**Figure 1:**
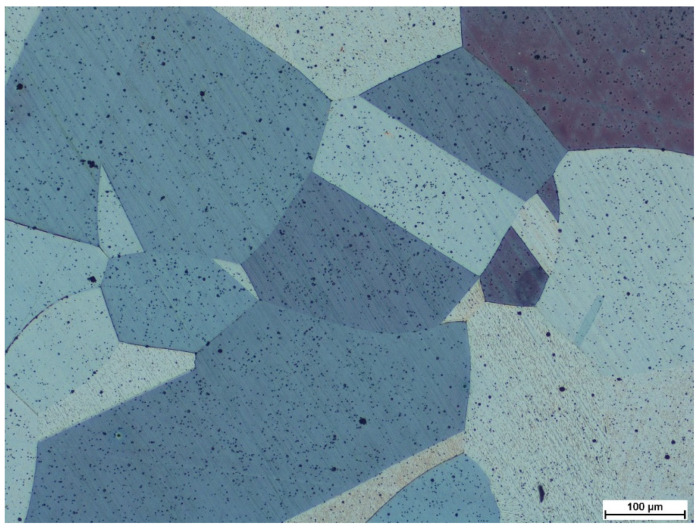
Material microstructure of the X120Mn12 steel in the as-delivered state. Microstructure of coarse-grained austenite with twins. Etched state. LM.

In terms of microstructure, the tested material has coarse austenite (average grain area of austenite $\bar{A}=48,409{\;\mu m}^{2}$, G = 1.5 grain size acc. to ASTM E112-13) with a trace amount of residual undissolved (during the process of oversaturation) globular carbides, which are spread evenly in the entire cross-section of the grains (Figure 1). This is a typical morphology for supersaturated and recrystallised Hadfield steel after plastic deformation processing. At the same time, they also lack the continuous carbide precipitations along the grain boundaries that could form a strength-reducing network. The size of the observed grains is mainly due to the casting processes and high temperature heat treatments. However, in contrast to the as-cast state, the tested material is free of any inhomogeneity of its chemical composition, or the presence of a dendritic structure, cracks, or porosity, which positively influences an increase of strength and a reduction of brittleness [35].

### 3.2. Analysis of the Material after Heat Treatment

#### 3.2.1. Identification of Microstructural Components

**Figure 2:**
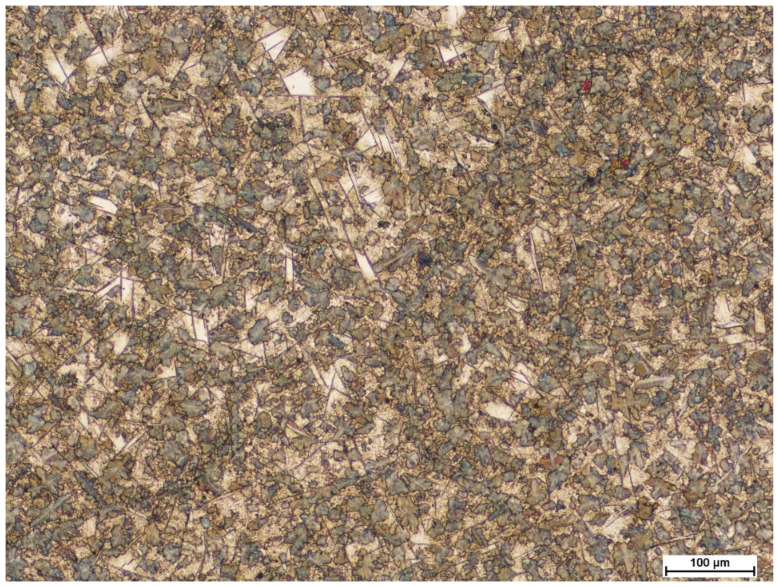
Microstructure of the X120Mn12 steel after isothermal annealing at 510 °C and cooling with the furnace. Visible diversified and refined microstructure morphology compared to the as-delivered condition (Figure 1). Etched state. LM.

**Figure 3:**
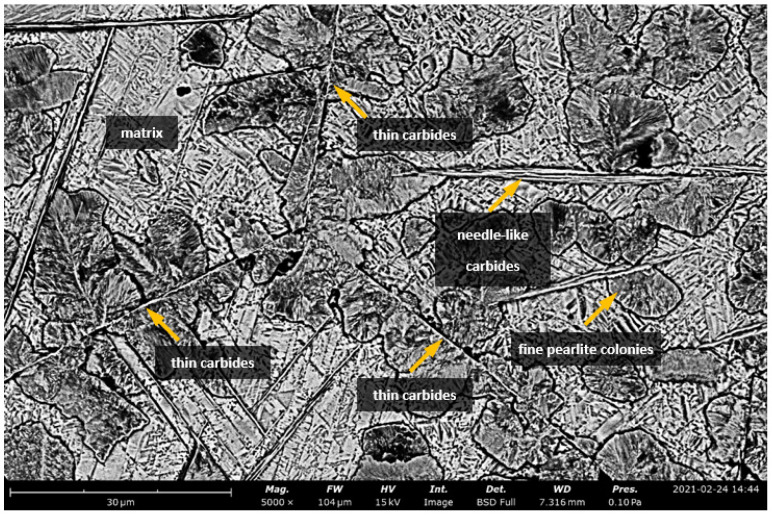
Microstructure of the X120Mn12 steel after isothermal annealing at 510 °C and cooling with the furnace. Visible heterogonous growth of new pearlite grains and needle-like precipitations at the grain boundaries of the former austenite, which are also the site of the nucleation of “thin” carbides. Etched state. SEM (BSE).

**Figure 4:**
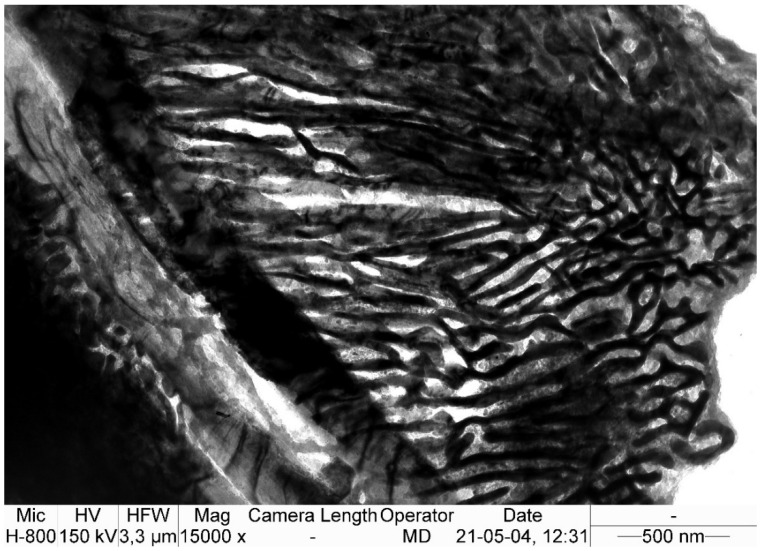
Microstructure of the X120Mn12 steel after isothermal annealing at 510 °C and cooling with the furnace. Brightfield image of a “thin” carbide about 0.35-μm thick at the grain boundary of the former austenite and a fine pearlite colony growing on it. TEM.

After isothermal annealing at 510 °C for twelve hours and subsequent cooling with the furnace until an ambient temperature was reached, the steel exhibited a diversified and refined microstructure morphology consisting of carbides of different structures and distributions, as well as numerous colonies of fine lamellar pearlite (average grain area of pearlite colonies: $\bar{A}=53{\;\mu m}^{2}$, G = 11.5 grain size acc. to ASTM E112-13) (Figure 2 and Figure 3), which was confirmed by transmission electron microscopy (Figure 4).

Heterogeneous grain growth and carbides were observed at the former austenite grain boundaries (Figure 2). An analogous distribution and size of carbides were found, as was the case in the work of S. Kuyucak [36]. The “thin” carbides that were separated along the grain boundaries (indicated by arrows in Figure 3), and the “thick” carbides (with a needle-like structure) that later grow on them and reach a length of up to 50 μm, can be distinguished. This, according to the results of the studies in [37], is associated with a low silicon content. There is a noticeable difference in the thickness of the two types of carbides. The thin ones form a continuous network of precipitations with a thickness of about 0.35 μm, while the thicker ones are characterised by a width ranging from 0.5 μm to 2.0 μm, which represents higher values with respect to the results of the study presented in the work of S. Kuyucak [36]. Presumably, this is due to the process of prolonged and isothermal annealing, which results in an increase in diffusion time and, ultimately, the thickening of the carbides. At the same time, the authors Kuyucak, Zavadil, and Gertsman suggest that thin carbides that are precipitated along the grain boundaries of former austenite pose less threat to the embrittlement of high-manganese steels than needle-like “thicker” carbides [36]. They hypothesisa that the crucial factor is the coherence of the matrix with the carbides at the grain boundaries, which the “thicker” carbides lack.

**Figure 5:**
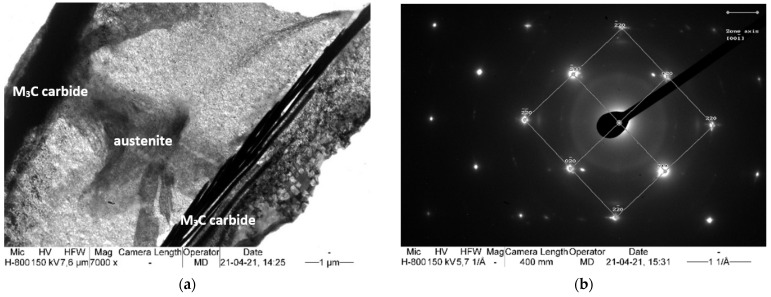
(**a**) Thin M_3_C carbide plates in the austenite matrix. (**b**) Diffraction pattern obtained from the grain of austenite with zone axis [$00\bar{1}$] showed in (**a**). TEM.

**Figure 6:**
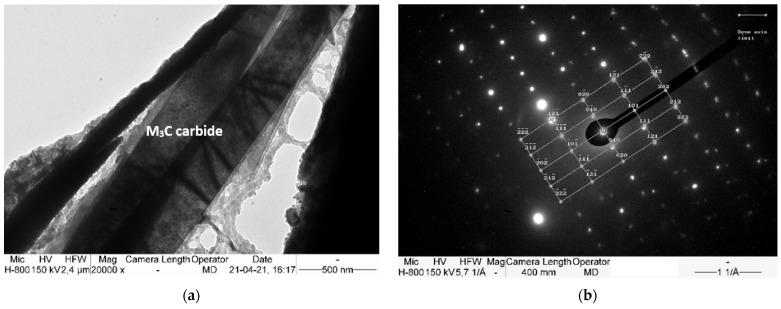
(**a**) Thin M_3_C carbide plates in the austenite matrix. (**b**) Diffraction pattern obtained from (Fe,Mn)_3_C carbide with zone axis [$\bar{1}01$] showed in (**a**). TEM.

**Figure 7:**
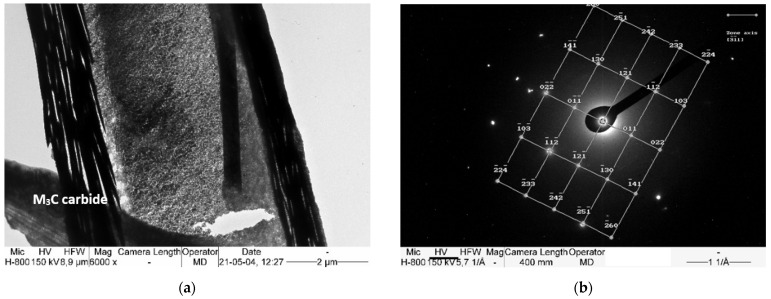
(**a**) Thin M_3_C carbide plates in the austenite matrix. (**b**) Diffraction pattern obtained from (Fe,Mn)_3_C carbide with zone axis [$\bar{3}\bar{1}1$] showed in (**a**). TEM.

Transmission electron microscope observations and the SAED method were used to determine the microstructural components after isothermal annealing. Figure 5, Figure 6 and Figure 7 show the areas subjected to diffraction, and also its results with the overlaid solution.

The carbide precipitations and fine pearlite colonies are found in the matrix, which is identified as austenite (Figure 5). No non-diffusion transformation components, such as martensite, were found. Such components could be formed as a result of the significant carbon depletion of the matrix, which is caused by the precipitations of an excessive amount of carbides.

All the identified carbides correspond to the M_3_C cementite lattice, but their chemical composition is not entirely evident. In addition, manganese carbide also has comparable lattice parameters [31]. For this reason, the most likely carbides to undergo SAED are manganese cementite. The presence of carbide with the stoichiometric formula (Fe,Mn)_3_C in high manganese steels is confirmed in many works [37,38,39,40,41,42], some of which mention the complexity of this carbide, which also contains chromium and/or vanadium [41]. The carbides take the form of a packet of needle-like precipitates, and have a total thickness of between 0.5 and 2.0 μm.

#### 3.2.2. Distribution of Chemical Elements

**Figure 8:**
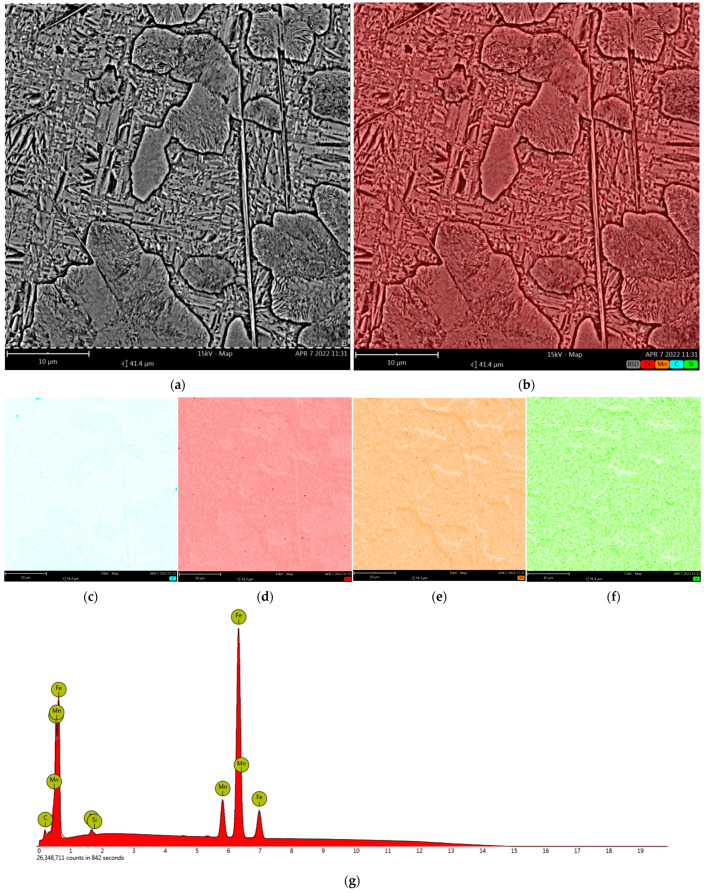
EDX surface microanalysis results: (**a**) area of analysis; (**b**) graphical distribution of the analysed elements; (**c**–**f**) the individual elemental distributions arranged sequentially as C, Fe, Mn and Si; (**g**) the energy spectrum of the X-rays obtained from the area analysed. SEM (EDS).

**Figure 9:**
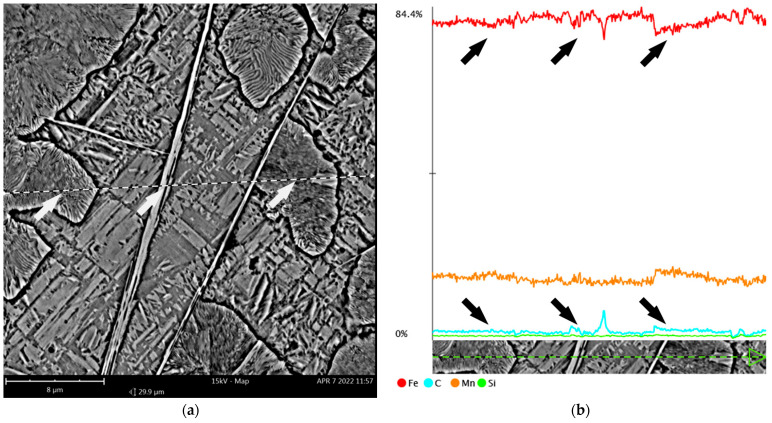
EDX linear microanalysis results: (**a**) the area of analysis with the line marked; (**b**) linear distributions of the individual elements Fe, Mn, C and Si. A visible local increase in carbon content in fine pearlite colonies and needle-like precipitates of (Fe,Mn)_3_C carbides (indicated by arrows). SEM (EDS).

According to the graphical composition of the elemental distributions obtained by the EDX surface microanalysis, there was no clear segregation of the key elements, i.e., C, Mn, and Si (Figure 8, Table 2). However, a slight increase in carbon content and a simultaneous decrease in iron content can be observed for the needle-like carbide precipitates (Fe,Mn)_3_C and fine pearlite colonies (Figure 8c,d). This was also confirmed during a linear microanalysis of the area shown in Figure 9, which was performed several times to increase accuracy.

#### 3.2.3. Hardness Measurements

**Figure 10:**
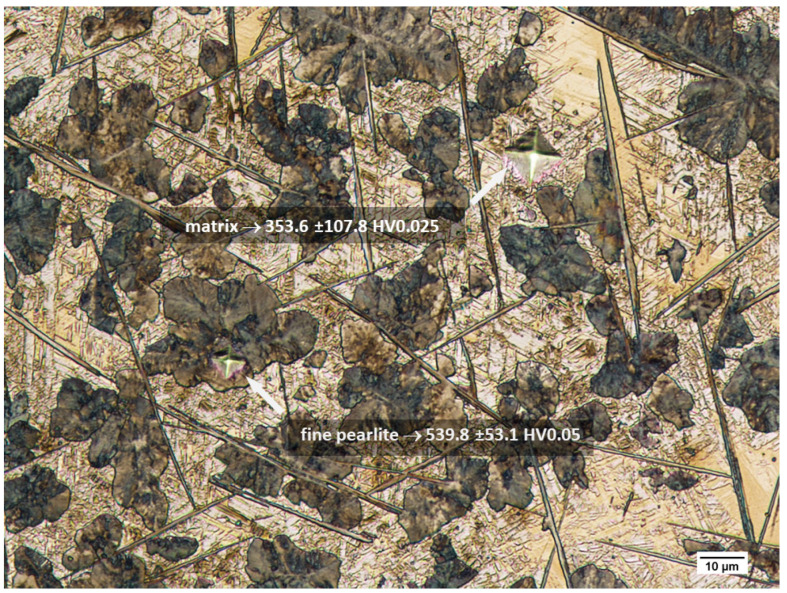
Microstructure of the X120Mn12 steel after isothermal annealing at 510 °C and cooling with the furnace, along with the determined microstructure components subjected to the microhardness measurements. Etched state. LM.

The results of the hardness measurements of the microstructural components making up the steel material after isothermal annealing showed that austenite was characterised by a hardness of 353.6 ± 107.8 HV0.025, which indicates the resulting strengthening of the matrix as a result of the heat treatment. Fine pearlite colonies are characterised by a hardness of 539.8 ± 53.1 HV0.05. The size of M_3_C carbides limits the possibility of measuring their hardness with a classic microhardness tester (Figure 10).

**Figure 11:**
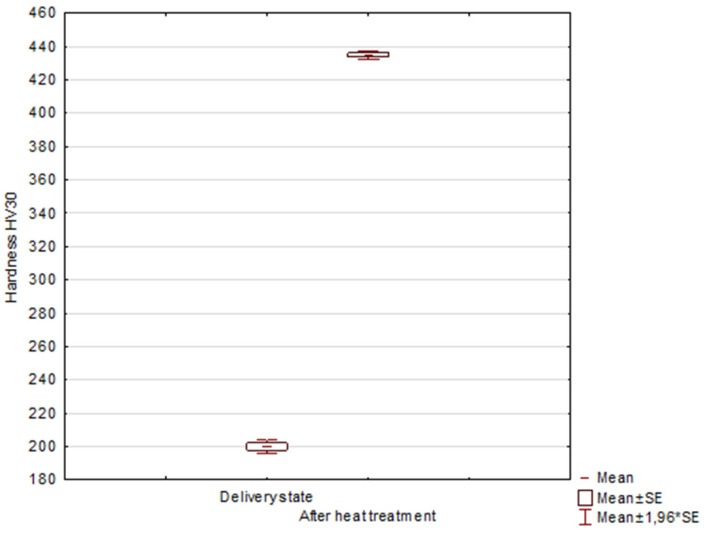
Box-and-whisker plot showing the significant effect on the increase in hardness values of the steel after heat treatment.

The material after isothermal annealing shows an average hardness of 435.0 ± 4.2 HV30, which, with respect to the measurements in the as-delivered condition of 200.0 ± 6.7 HV30, illustrates the significant effect of the alternative heat treatment on the increase in the material hardness (*p* < 0.01; Student’s *t*-test for α = 0.05) (Figure 11).

### 3.3. Re-Saturating Heat Treatment (Re-Austenitisation)

**Figure 12:**
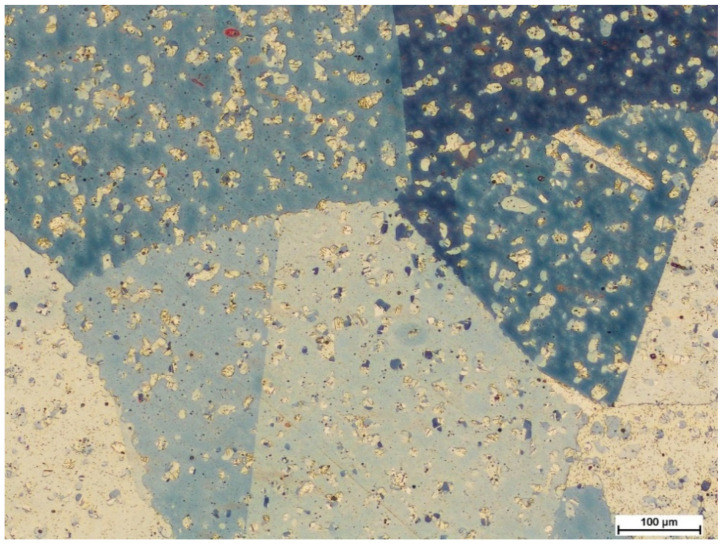
Microstructure of X120Mn12 steel after re-austenitisation at 900 °C. Sample previously isothermally annealed at 510 °C and cooled with furnace. Significant disappearance of the amount of carbides and fine pearlite colonies from the previous state is visible. Numerous new austenite grains of less than 20 μm in size are visible. Etched state. LM.

**Figure 13:**
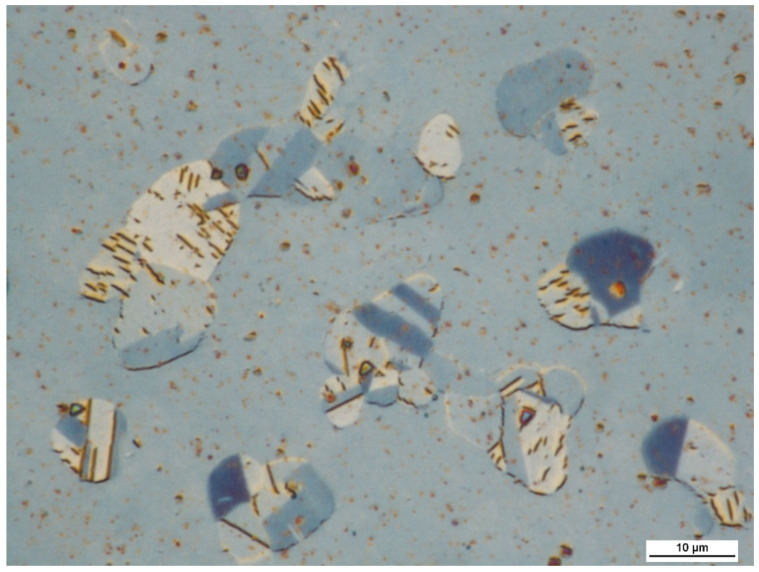
Microstructure of X120Mn12 steel after re-austenitisation at 900 °C. Sample previously isothermally annealed at 510 °C and cooled with furnace. Numerous new austenite grains of less than 20 μm in size can be seen with characteristic twin grains boundaries and fine, evenly distributed carbide precipitations. Etched state. LM.

According to many of the results of the scientific work presented in the introduction of this paper, Hadfield steel should be used in a supersaturated, carbide-free state. For this reason, the previously isothermally annealed steel was experimentally re-saturated at 900 °C, which is lower than that recommended in works [1,39] or in patent [3]. As a result, the microstructure of the steel was considerably finer grained than in the as-delivered condition, devoid of the previously observed M_3_C carbides at the grain boundaries of the former austenite, and in the form of needles. Furthermore, no previously numerous fine pearlite colonies were visible (Figure 12). Within the original austenite grains (with G = 1.5 grain size acc. to ASTM E112-13), and also at their boundaries, numerous new austenite grains (smaller than 20 μm with characteristic twinned boundaries) were visible (Figure 13). The percentage of new grains in the analysed sample is 21.3% and their size is G = 11.5 acc. to ASTM E112-13, which corresponds to average grain area $\bar{A}=49.8{\;\mu m}^{2}$. M_3_C carbides and fine pearlite colonies were used as the substrate for heterogeneous nucleation of new grains, which resulted in the fragmentation of the originally coarse-grained microstructure of the X120Mn12 steel (particle stimulated nucleation). At the same time, the austenite matrix exhibited numerous, evenly distributed M_3_C carbides with a globular shape, which ultimately resulted in the formation of a heterogeneous microstructure with potentially increased resistance to abrasive wear compared to the as-delivered or cast state. The material after the re-austenitisation process has a hardness of 228.5 ± 6.4 HV30, which represents an increase in the hardness of the material in relation to the as-delivered condition as a consequence of grain refinement of the microstructure and the remains of undissolved M_3_C carbides. At the same time, there is a visible decrease in the hardness of the material with respect to the state after isothermal annealing. This is due to the dissolution of a high percentage of carbides and pearlite colonies.

### 3.4. Comparative Abrasive Wear Resistance Tests

**Figure 14:**
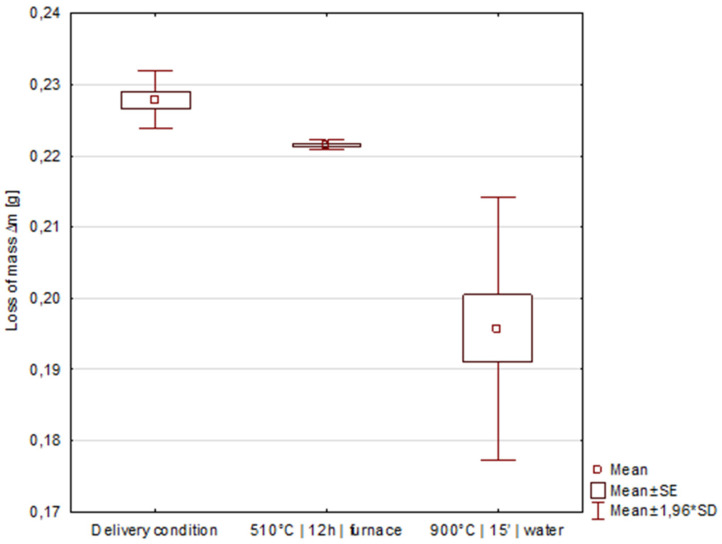
Box-and-whisker plots showing a significant effect (*p* < 0.01; ANOVA NIR test for α = 0.05) on the increase in abrasive wear resistance of X120Mn12 steel relative to the delivery condition achieved by the modified heat treatment process.

As a result of the experiment, it is demonstrated that the phenomena of precipitation strengthening with globular (Fe,Mn)_3_C carbides and the boundaries of new austenite grains can be a mechanism to increase the abrasion wear resistance of commercially available X120Mn12 steel (Table 3). The two-stage heat treatment procedure (isothermal annealing followed by re-austenitisation) results in an increase in abrasion resistance compared to the delivery condition of approximately 16.4%, which was also verified by statistical tests (Figure 14). In the case of the condition after isothermal annealing, despite a significant increase in the hardness of the material, the increase in abrasion resistance is modest, amounting to 2.9% relative to the delivery condition. At the same time, the mass losses for the state after isothermal annealing are close to each other as a result of long-term annealing and cooling with the furnace, resulting in a homogenisation of the material microstructure due to diffusion processes and the attainment of a near-equilibrium state.

**Figure 15:**
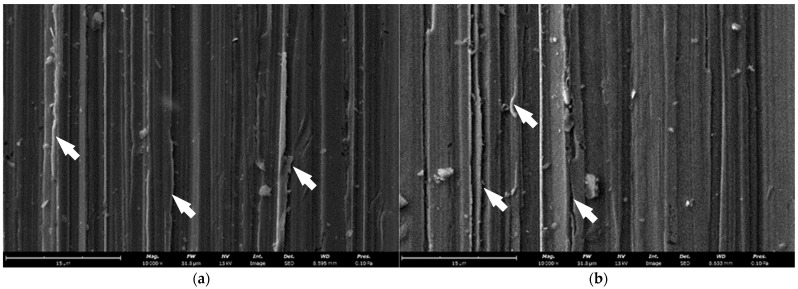

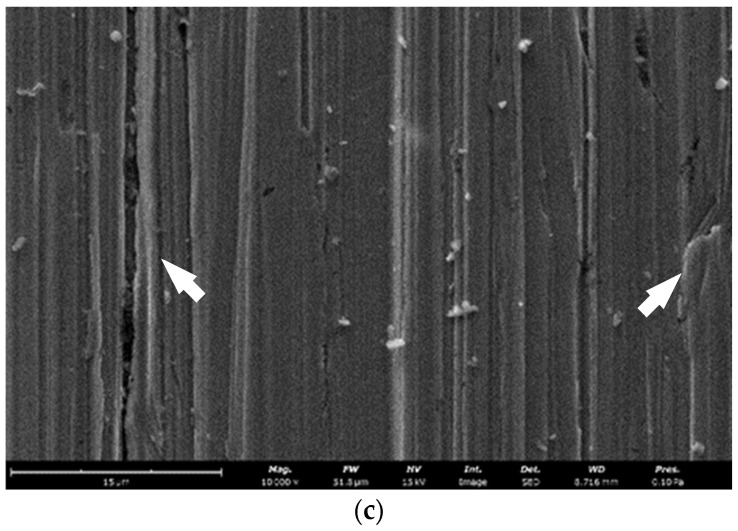
Morphology of the specimen surface after the abrasive wear test: (**a**) delivery condition; (**b**) after isothermal annealing at 510 °C; (**c**) after re-austenitisation at 900 °C (arrows indicate plastic deformation of the material caused by microploughing). SEM (SE).

The main wear mechanism is low-stress abrasion or scratching abrasion, in which hard particles groove and/or plough the surface of the material. Visible grooves longitudinal to the direction of friction are the effect of microcutting and microploughing, which confirms that the wear mechanism was abrasive in nature (Figure 15). The wear patterns are unidirectional with similar groove widths, which results from the nature of the test described in the methodology. At the same time, the character of wear is consistent for all samples, regardless of the microstructure state and hardness of the tested material.

## 4. Conclusions

On the basis of the tests performed on the X120Mn12 steel in the as-delivered condition, and the modified heat treatment in the form of a long-term isothermal annealing at 510 °C and a final re-saturation at 900 °C, the following conclusions were drawn:

1. The examined isothermally annealed steel is composed of four microstructural components: an austenitic matrix, (Fe,Mn)_3_C carbide in the form of a continuous network along the grain boundaries of the former austenite, (Fe,Mn)_3_C carbide in the form of needle-like precipitations, and fine pearlite colonies.
2. The hardness of the steel in the as-delivered condition was 200.0 ± 6.7 HV30, which after the isothermal annealing treatment increases in a statistically significant manner, when compared to the as-delivered condition, to 435.0 ± 4.2 HV30. This growth is due to the precipitation of (Fe,Mn)_3_C carbides and the formation of numerous fine pearlite colonies.
3. Final re-saturation at a lower than recommended temperature results in refining of the original coarse-grained microstructure of the steel. Furthermore, the morphology obtained is heterogeneous, and with a uniform and globular distribution of not fully dissolved carbide precipitations in the refined austenite matrix (Fe,Mn)_3_C, which resulted in a hardness of 228.5 ± 6.4 HV30, i.e., greater than in the as-delivered condition. The percentage of new grains in the re-austenised sample is 21.3% and their size is G = 11.5 acc. to ASTM E112-13 compared to the initial austenite grains with a size of G = 1.5. For this reason, the phenomenon of precipitation and the strengthening of the grain boundaries may be a mechanism to increase the abrasive wear resistance of X120Mn12 steel with an unmodified chemical composition without the addition of carbide-forming elements.
4. Comparative wear resistance tests carried out indicate that a two-stage heat treatment (isothermal annealing followed by re-austenitisation) results in a 16.4% increase in abrasion resistance compared to the delivered condition.

## Figures and Tables

**Table 1:** Results of spectral analysis of the chemical composition of the steel in the as-delivered state.

for *n* = 10 Replicates	C	Mn	Si	P	S	Cr	Cu	Mo	Ni	Al	Ti
average	1.04	12.5	0.404	0.023	0.000	0.230	0.151	0.019	0.077	0.025	0.006
standard deviation	±0.01	±0.1	±0.005	±0.000	±0.000	±0.004	±0.003	±0.000	±0.001	±0.001	±0.000

**Table 2:** The quantitative results of the surface microanalysis of the chemical composition shown in Figure 8.

Content	Mn	C	Si	Fe
at. [%]	11.81	11.05	0.95	bal.
wt. [%]	12.82	2.62	0.53	bal.

**Table 3:** Test results for abrasive wear resistance.

Samples		Heat Treatment Variants Subjected to Abrasive Wear Test		
		Delivery Condition	510 °C|12 h|Furnace	900 °C|15′|Water
Loss of mass ∆m * [g]	1	0.2256	0.2214	0.2066
	2	0.2297	0.2219	0.1917
	3	0.2282	0.2212	0.1850
	4	-	-	0.1998
	Average	0.2278	0.2215	0.1958
Relative resistance to abrasive wear in relation to delivery condition ** [–]		-	1.0286 (≈2.9%)	1.1638 (≈16.4%)

* The loss of mass was obtained by testing in accordance with GOST 23.208-79 using a T-07 tribotester. ** The abrasive wear resistance coefficient was calculated according to GOST 23.208-79 (due to the identical nature of the test material, the coefficient is the ratio of the weight loss of the test material to the weight loss of the reference material).

## Data Availability

Not applicable.
